# Lungfish Axial Muscle Function and the Vertebrate Water to Land Transition

**DOI:** 10.1371/journal.pone.0096516

**Published:** 2014-05-02

**Authors:** Angela M. Horner, Bruce C. Jayne

**Affiliations:** 1 Department of Biology, California State University, San Bernardino, California, United States of America; 2 Department of Biological Sciences, University of Cincinnati, Cincinnati, Ohio, United States of America; University of Utah, United States of America

## Abstract

The role of axial form and function during the vertebrate water to land transition is poorly understood, in part because patterns of axial movement lack morphological correlates. The few studies available from elongate, semi-aquatic vertebrates suggest that moving on land may be powered simply from modifications of generalized swimming axial motor patterns and kinematics. Lungfish are an ideal group to study the role of axial function in terrestrial locomotion as they are the sister taxon to tetrapods and regularly move on land. Here we use electromyography and high-speed video to test whether lungfish moving on land use axial muscles similar to undulatory swimming or demonstrate novelty. We compared terrestrial lungfish data to data from lungfish swimming in different viscosities as well as to salamander locomotion. The terrestrial locomotion of lungfish involved substantial activity in the trunk muscles but almost no tail activity. Unlike other elongate vertebrates, lungfish moved on land with a standing wave pattern of axial muscle activity that closely resembled the pattern observed in terrestrially locomoting salamanders. The similarity in axial motor pattern in salamanders and lungfish suggests that some aspects of neuromuscular control for the axial movements involved in terrestrial locomotion were present before derived appendicular structures.

## Introduction

The transition from water to land was a pivotal event in the evolution of vertebrates, and many of the associated morphological innovations, especially those of the appendicular structures, are now well documented from a rich fossil record [1]. Unlike limbs, the segmental axial muscles and skeleton and the sizeable post-anal tail in early tetrapods are all ancient features of chordates and vertebrates that were retained with little apparent modification. However, morphological conservatism need not preclude the evolution of functional novelty—alterations of neuromuscular patterns alone can result in novel behaviors. As such, studies of extant taxa can provide insights into ancient forms of locomotion in several ways. For example, extant taxa that are morphologically similar to extinct taxa are likely to have similar biomechanical constraints, and extant taxa that are closely related to an extinct group of interest often have some of the same basal features of an extinct form. Furthermore, extant groups with ecological niches similar to those of extinct forms may be placed into putatively ancestral or derived environments to determine how locomotor strategies may vary with changes in habitat.

Salamanders are widely-used morphological analogues for early tetrapods, and they employ distinctly different axial movements and neuromuscular patterns while moving in terrestrial versus aquatic environments [2]–[5]. However, some primarily aquatic organisms such as eels and rope fish occasionally move on land and do so with axial movements [6] and muscle activity [7], [8] that are grossly similar to the patterns used when swimming in water, including a posteriorly propagated wave of bending and motor activity, or ‘traveling’ wave.

Swimming with traveling waves of lateral bending in the vertebral column is an ancient, shared trait, present in such phylogenetically diverse vertebrates as agnathans, cartilaginous and bony fishes, amphibians, and elongate reptiles. Furthermore, the aquatic axial motor patterns of these taxa share major features such as muscle activity that propagates posteriorly along the length of the animal and rhythmically alternates between the left and right sides at a given longitudinal location [4], [9]–[11]. Many of these same taxa are periodically terrestrial but lack substantial appendicular structures, and so axial structures must be used for both aquatic and terrestrial locomotion. This is the case for eels, which use traveling waves of axial bending and muscle activity in both water and on land [7]. Limbed salamanders also swim with posteriorly propagated waves of axial bending and muscle activity, but they trot on land utilizing standing wave patterns in their trunk, with large numbers of adjacent ipsilateral axial muscle segments simultaneously active [3], [4]. These observations suggest that the presence of limbs was associated with a shift from a traveling to a standing wave of axial muscle activity during terrestrial locomotion, such that the bending of the trunk serves to extend the stride length of relatively simple limbs Whereas the occurrence of propagated, traveling waves of muscle activity is a widely documented feature of the locomotion of many vertebrates, standing wave motor patterns during locomotion have not been observed in fish except for the very fast, transient **C**-start escape response.

Among aquatic vertebrates that are occasionally terrestrial, the lungfish clade (Sarcopterygii: Dipnoi) is of particular interest because they are the sister taxon to modern tetrapods [12], [13], and can move on or through muddy media with a large variety of viscosities and firmness [14], [15]. Unlike their known fossil ancestors and transitional tetrapods such as *Tiktaalik roseae* [16], five of the six extant lungfish species (including the African lungfish) have secondarily derived diminutive paired fins and a uniformly cylindrical trunk that resembles other elongate, aquatic vertebrates. Although the slenderness of the fin appendages precludes any significant weight-bearing, a previous study of African lungfish [17] demonstrated that tetrapod gait patterns were evident in the movement of the paired fins in a fully aquatic environment. Limbs and gaits aside, most of the muscle mass of these fish and fish-like descendants occurs in the axial portion of the body, and thus the movement of the axial structures is of key importance to understanding the evolution of terrestrial locomotion.

In a previous study on the effects of viscosity on axial function during swimming, we collected EMG and kinematic data from the African lungfish (*Protopterus annectens*) from a longitudinal array of electrodes [15]. When African lungfish swim through a more viscous medium, they use axial movements and muscle activity that are similar to those used during swimming in water, even in a viscosity 1000 times greater than water—although there were differences in the timing of flexion relative to muscle activity, locomotion in all viscosities was powered by a posteriorly-directed traveling wave of axial muscle activity. However, it is unclear whether or not this traveling wave axial motor pattern is conserved across a more pronounced, discrete shift in environment such as in the transition from water to land. Therefore, in the current study, we measured axial movements and muscle activity during the terrestrial locomotion of the same species of African lungfish to test for novelty versus continuity of axial movement and motor pattern associated with an acute change in environment. The elongate body and reduced appendages of lungfish suggest that the terrestrial locomotion of lungfish should resemble that of morphologically similar organisms such as eels, which retain many features of an aquatic motor pattern. However, the diagonal couplet pattern of fin movements that has been observed in cartilaginous fishes, coelacanths, and lungfish [17]-[20] suggests that some aspects of terrestrial motor patterns may have evolved before limbed terrestrial locomotion, and thus lungfish may behave more tetrapod-like whilst on land. Here we evaluate the kinematics and motor pattern of the terrestrial locomotion of lungfish and compare our findings to the previous study of lungfish swimming in aqueous media, as well as to the trotting of salamanders.

## Materials and Methods

### Electromyography

**Figure 1:**
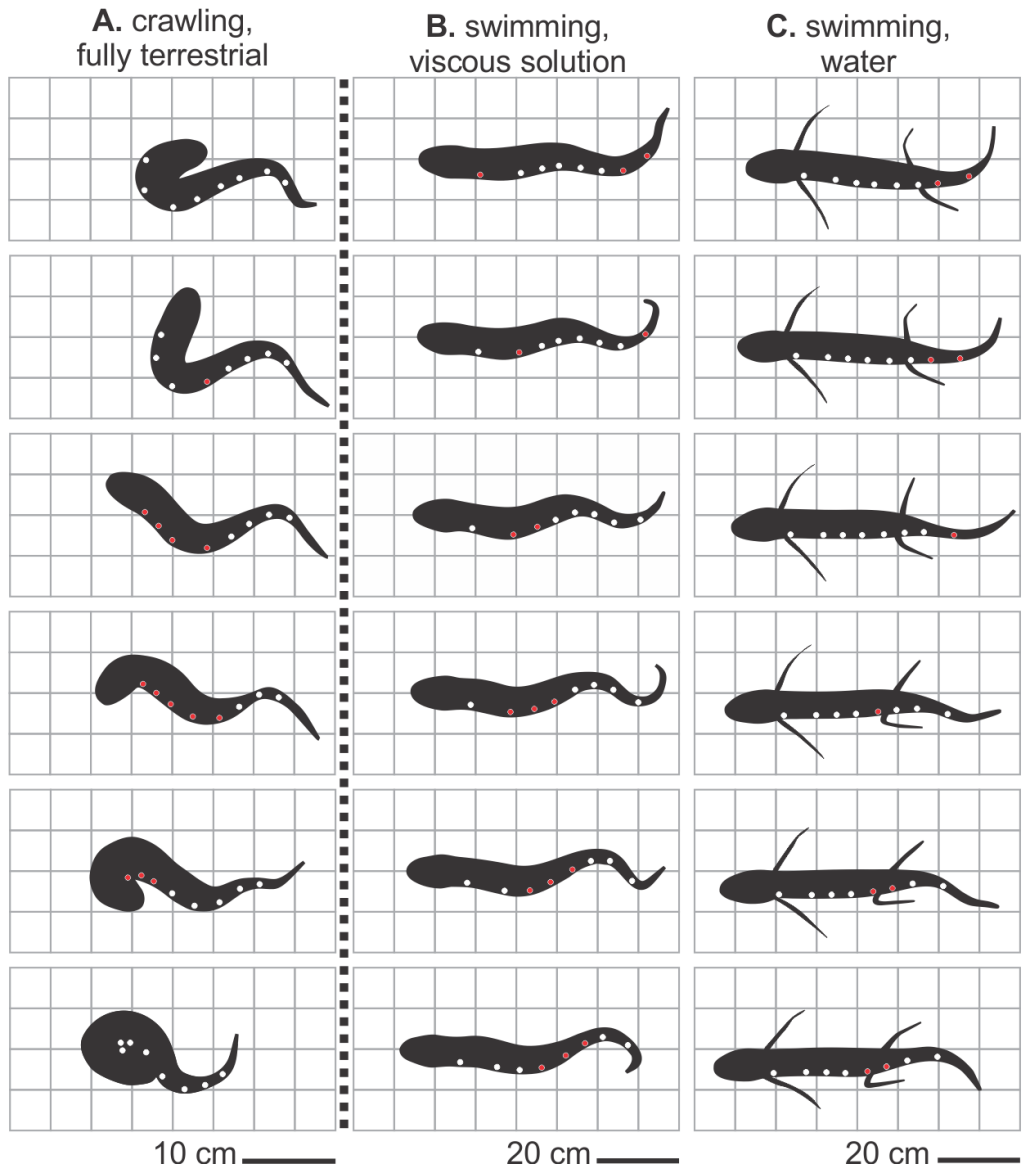
Dorsal images from videos of lungfish locomotion. These images represent one-half cycle of locomotion on a firm mud surface (*A*), and for swimming in aqueous media (*B* and *C*; ventral view, modified from [15]). Time progresses from the top to bottom in each panel. Sites for EMG electrodes are denoted with circles; red and white indicate activity or no activity, respectively. In *B* and *C*, the lungfish were swimming in aqueous media with viscosities of 1000 cSt and 1 cSt, respectively. During terrestrial locomotion, multiple adjacent trunk EMG sites are active whilst the trunk is bending. By contrast, during swimming both waves of muscle activity and bending are posteriorly propagated (‘traveling’).

Following the procedures described in more detail in Horner and Jayne [15], we used eleven bipolar fine wire electrodes (0.051 mm diameter) to record electromyograms (EMGs) of the axial muscles at eight homologous longitudinal locations (Fig. 1) spaced evenly along the length of African lungfish, *Protopterus annectens* (Owen 1839). For example, sites 1–5 and 6–8 were in the trunk and caudal regions, respectively, and each site corresponded to a similar percent total length (Table 1). On the left side of the fish electrodes were placed in superficial red muscle at sites 1–8; on the right side of the fish electrodes were placed in superficial red muscle at sites 2, 4 and 7. These sites were only used to confirm that activity was unilateral, and thus were not used in the statistical analysis. We implanted electrodes in four lungfish, but due to the tendency of the lungfish to burrow during the experiments, the electrodes were often dislodged or damaged during the course of experiments and our quantitative analysis of data was necessarily confined to the two individuals with the most complete longitudinal coverage of electrodes; the total lengths of these two individuals were 28 and 32 cm. During experiments the lungfish were placed into a 284 L aquarium with a layer of wet sculptor's clay (1∶1 dry clay to water ratio) spread evenly on the bottom, and the temperature of the mud surface was at 24±1°C. All procedures were approved by the University of Cincinnati Institutional Animal Care and Use Committee (protocol 07-01-08-01).

**Table 1:** EMG implantation sites in lungfish as a fraction of total length (*L*).

	Total Length (*L*)	
Longitudinal site	Individual 1	Individual 2
**1**	0.17	0.19
**2**	0.24	0.26
**3**	0.32	0.35
**4**	0.39	0.44
**5**	0.49	0.54
**6**	0.57	0.62
**7**	0.64	0.74
**8**	0.75	0.83

The analog EMGs were recorded with a TEAC XR-5000 FM data recorder; we used a 100 Hz square-wave transmitted simultaneously to both the video system and the TEAC data recorder to synchronize the video and EMGs. After the experiments the positions of the electrodes were confirmed by post-mortem dissections and radiographs. We converted analog EMGs to digital data using an AD Instruments 16SP analog-to-digital converter and software (Chart version 5) with an effective sampling rate of 8.8 kHz and a 50 Hz high-pass filter.

For the digital EMGs, we measured the times of onset, offset, and duration (offset – onset) for each burst of muscle activity. Variables describing the time course of events were converted to a relative time scale by standardizing them so that the onset of the most anterior EMG was time zero, and elapsed times ranged for 0-100% of the duration of a locomotor cycle. The temporal overlap of ipsilateral EMGs was quantified such that a pair of EMGs from different longitudinal locations with identical times of onset and offset would have a value of 100% overlap, which would indicate strict conformity to a standing wave pattern. We quantified the percent overlap of EMGs from the trunk region (sites 1–5) to facilitate comparisons with previous data from terrestrial salamander locomotion. We only analyzed EMGs from electrodes that remained in place throughout the entire experiment and recorded a total of 25–30 cycles of terrestrial undulations per fish.

### Kinematics

Out of over 40 trials from the two individuals, we analyzed a subset (N = 19) of these that were characterized by 1) symmetrical direction of travel, and 2) steady bouts that did not include pauses. A dorsal view of the lungfish was videotaped via a mirror angled at 45° using a NAC HSV 500 camera operating at 250 images s^−1^. For approximately 200 equally-spaced time intervals within each locomotor cycle, the two-dimensional locations of twenty points evenly spaced along the dorsal midline of a fish were digitized in Didge Image Digitizing Software [21]. The distances between successive midline points spanned three to five vertebrae. Angles of lateral flexion were calculated for the three midline points nearest each electrode site.

### Statistical analyses

We performed statistical analyses using SYSTAT v. 12.0. Of the eight superficial ipsilateral EMG sites, seven were used for statistical analyses (site 6 was excluded due to electrode displacement in one individual). We performed 2-way, mixed-model ANOVAs and treated individual as a random factor, and longitudinal site or region (i.e., trunk vs. tail) as a fixed factor.

## Results

### Kinematics

The terrestrial locomotion of the lungfish on mud involved stereotyped, regular progression of events (Fig. 1A; Video S1); after moving the head slightly to one side, the lungfish ventrally flexed the anterior trunk so that the head dug slightly into the mud surface, leaving evenly spaced impressions into the mud. As the trunk region continued to flex tightly to one side, the tail was drawn closer to the head while the head made little if any forward progress. As the body of the fish straightened, the head often moved forward rapidly while the tip of the tail often made little forward progress. Similar events then repeated for the contralateral side. Movements of the tail were often irregular, and hence the timing of lateral flexion of the tail did not correspond to that of the trunk region in a predictable manner. Unlike swimming in either water (Fig. 1C) or a highly viscous solution (Fig. 1B), the terrestrial locomotion of lungfish (Fig. 1A) had maximum angles of lateral flexion in the trunk region (36±1.4°) that were significantly greater (F = 1,195.0, d.f. = 1,1, *p* = 0.02) than those for the caudal region (trunk, 23±1.5°). The lateral displacements and flexion during terrestrial locomotion were considerably larger than those observed for any type of swimming (Fig. 1).

### Axial muscle activity

**Figure 2:**
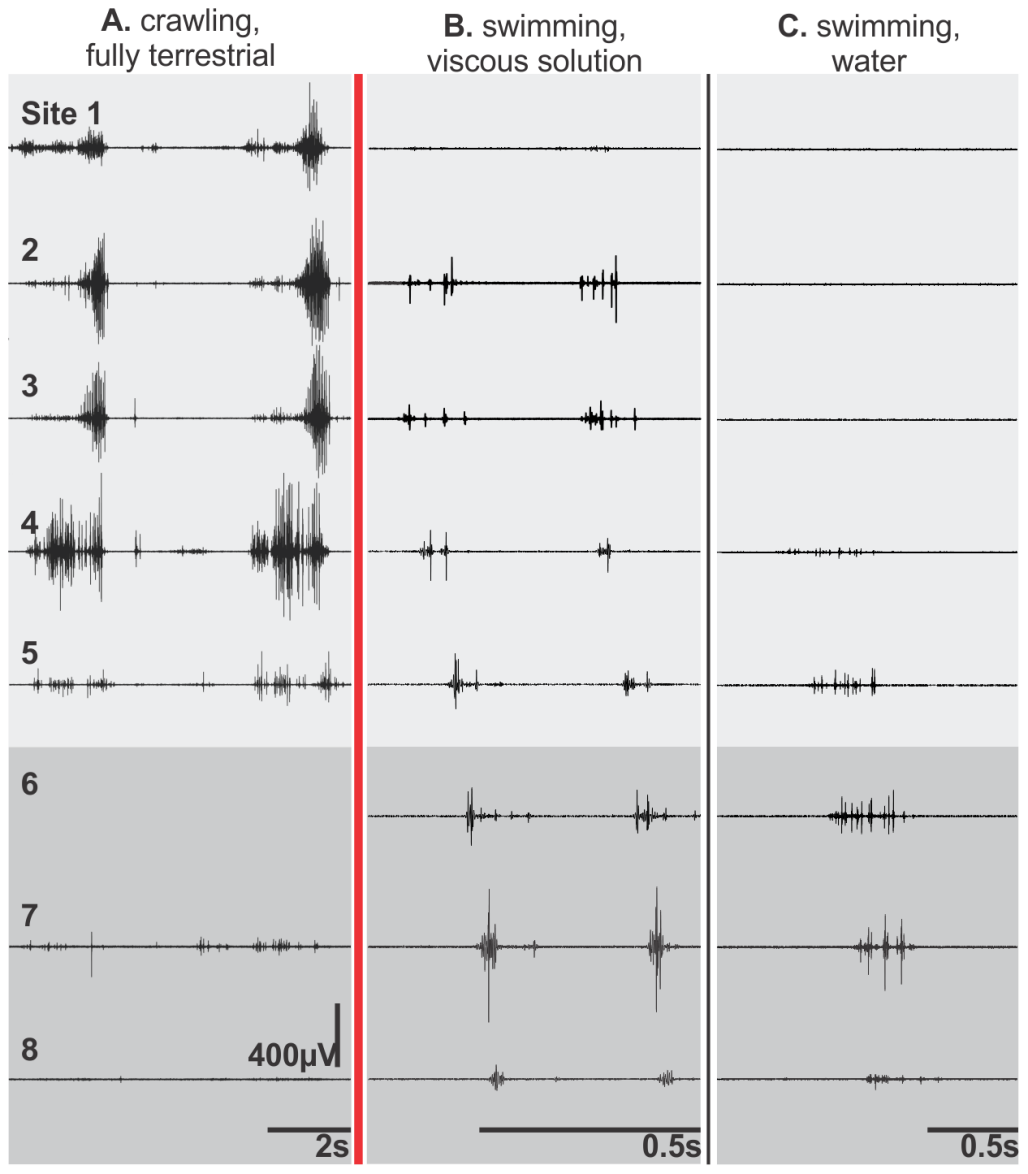
Representative EMGs from lungfish locomotion in different media. Motor patterns are compared among three conditions: fully terrestrial locomotion on mud (*A*), swimming in a very viscous solution (B), and in water (*C*; modified from [15]. Sites 1–5 (light shading) and 6–8 (dark shading) are from myomeres in the trunk and tail, respectively. EMGs during terrestrial locomotion were higher in amplitude, active for a greater proportion of a cycle, restricted mainly to the trunk region, and active almost simultaneously. Conversely, lungfish swimming is powered primarily by caudal muscle activity that is propagated posteriorly. For the sequence shown in panel A, the electrode at site 6 was dislodged.

**Figure 3:**
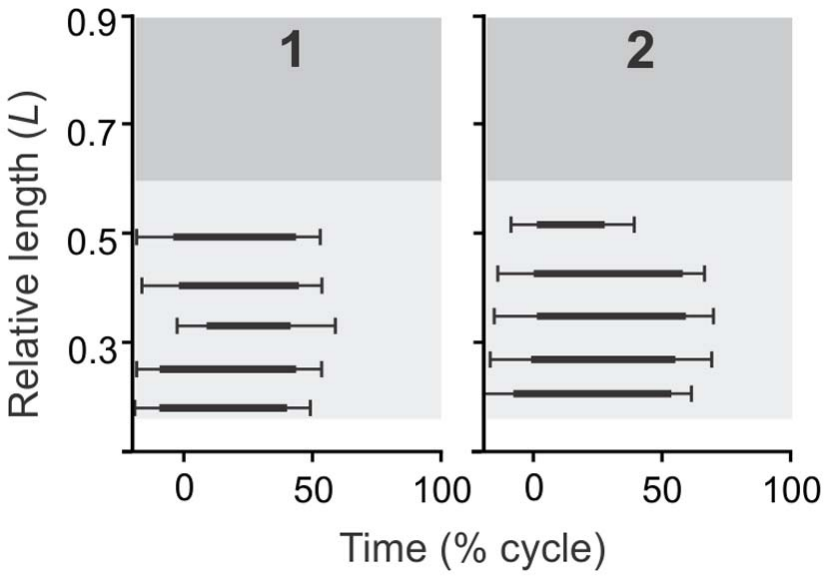
Comparison of mean EMG activity during terrestrial locomotion by longitudinal site between two lungfish. Error bars are standard deviations of onset and offset timing. All times are expressed as fractions of a locomotor cycle (x-axis), and longitudinal locations (y axis) are the distance from the snout of the animal expressed as a proportion of its total length (*L*). Dark shading, light shading, and white indicate locations in the tail, trunk region and anterior to the pectoral girdle, respectively (head excluded).

**Figure 4:**
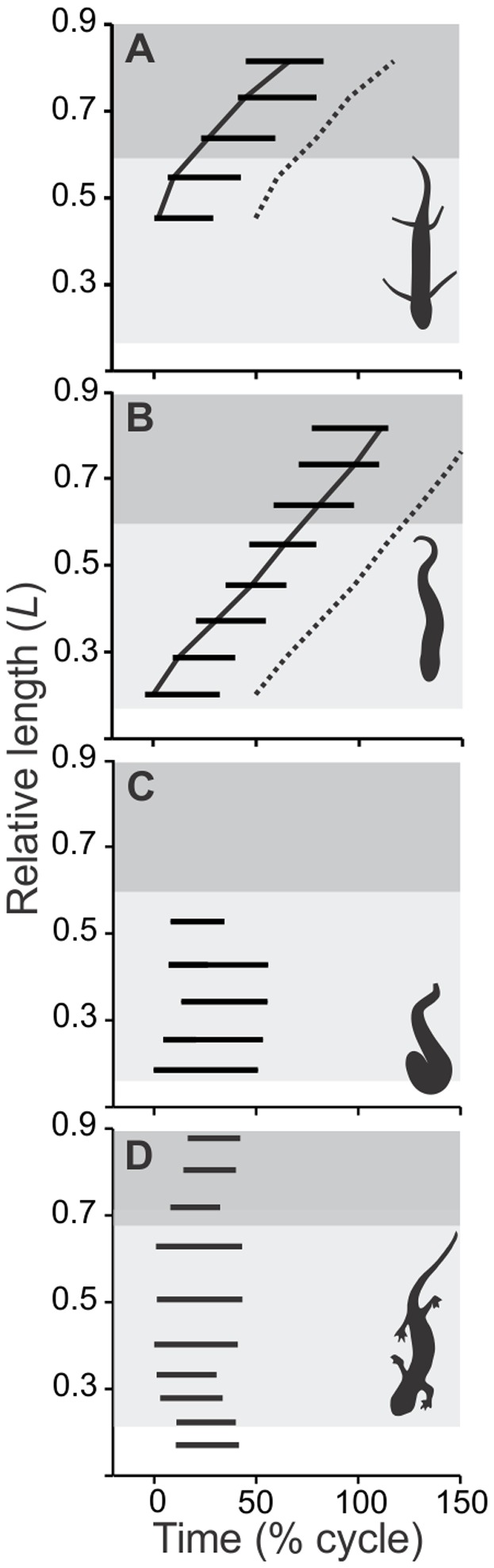
Summaries of mean values for the timing of epaxial EMGs (thick horizontal bars) among four different types of locomotion. Axes and shading follows the convention used in Fig. 3. We compared the mean motor pattern and bending of lungfish during terrestrial locomotion (*C*; this study) to lungfish swimming in water and a high viscosity medium (A and B; modified from [15]), and salamanders stepping on land (D; modified with permission of authors [23]). Swimming lungfish (*A* and *B*) demonstrate a posteriorly propagated motor pattern, with caudal sites active nearly half of a locomotor cycle later than anterior sites. Terrestrial lungfish and salamanders (*C* and *D*), however, show near simultaneous onsets of muscle activity bursts.

**Figure 5:**
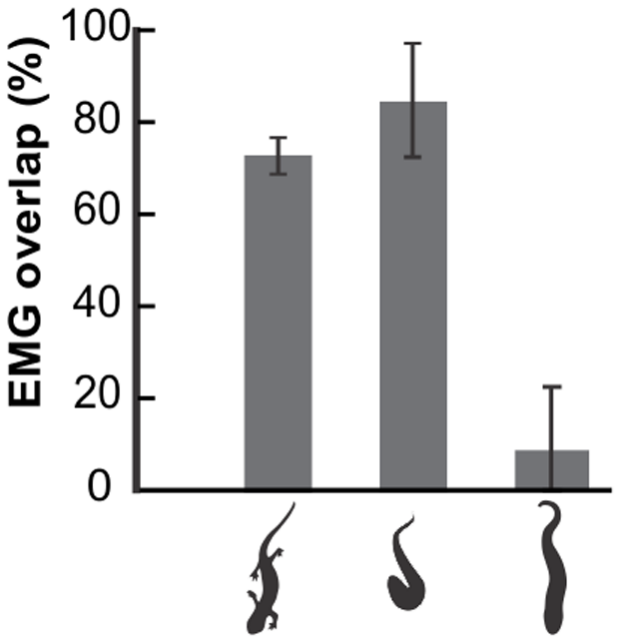
Mean (± s.e.m) percentages of temporal overlap of epaxial muscle activity among trunk EMG sites of salamanders and lungfish moving in different environments. Refer to sites in the light gray shaded box in Fig. 3 for details of sampling extent for each taxon or substrate. From left to right, the values are for a salamander walking on land (used with permission of[23]), a lungfish crawling on a firm mud surface, and swimming in a 1000 cSt solution [15]. The terrestrial locomotion of both salamanders and lungfish had extensive overlap, indicating that muscle activity in the trunk region best resembles a standing wave pattern, whereas the propagated motor pattern during the swimming of lungfish resulted in nearly no overlap in the timing of muscle activity spanning four longitudinal sites.

The EMGs of superficial ipsilateral sites in the trunk region during terrestrial locomotion were nearly synchronous, whereas swimming lungfish clearly had posteriorly propagated (traveling) waves of muscle activity (Figs 2–4). During terrestrial locomotion, EMGs from the most anterior (site 1) and most posterior (site 5) locations in the trunk region overlapped an average of 85.0±11.9% (Fig. 5). By contrast, ipsilateral EMGs in the trunk region of lungfish overlapped less than 10% for swimming, even in a very viscous solution (6.4±4.6%; Fig. 5). For all types of lungfish locomotion, none of the pairs of EMGs from opposite sides at the same longitudinal location had overlapping activity, and activity rhythmically alternated between the left and right sides as has been widely described for other vertebrates that move *via* lateral undulations.

Within an individual, the longitudinal location significantly affected the intensity of muscle activity (2-way ANOVA; *F* = 6.21, d.f. = 1, 6, *p*<0.05). During terrestrial locomotion, large amplitude EMGs were consistently detected in trunk sites, but EMGs in the caudal sites were irregular, low amplitude, and frequently entirely absent (Fig. 2). Even allowing for variation among electrodes and between individuals, this differs substantially from the general observed during aquatic swimming. During slow, steady swimming in water, EMGs were only observed consistently in the caudal sites (Fig. 2C), though in progressively greater viscosity the longitudinal extent and amplitude of EMGs during swimming increased anteriorly to involve trunk sites (Fig. 2B). During terrestrial locomotion, trunk EMGs had burst durations that averaged approximately one half (51±11%) of a cycle, with onsets very near when the body was maximally convex and offsets very near the time when the body was maximally concave on the side with muscle activity (Fig. 3). Among active sites in lungfish swimming in water and the high viscosity medium, burst durations were 36±2% and 35±1% of a cycle, respectively, and the muscle activity usually ceased well before the body was maximally concave on the side of muscle activity (Fig. 4).

## Discussion

### The terrestrial locomotion of lungfish vs. aquatic locomotion

The axial muscle activity of lungfish during terrestrial locomotion was not merely one end of a continuum of variation as in lungfish swimming in fluids with increasing viscosity [15]. Instead, movement on land shifted lungfish from tail-driven to trunk-driven propulsion, and the longitudinal timing of muscle activity during terrestrial locomotion was nearly synchronous, rather than propagated. Previous data from eels [7], [8], lungfish [15], and ropefish [6] initially suggested to us that the locomotion of lungfish on land would share some gross features of swimming, but differ in the details related to timing and magnitude. For example, the terrestrial locomotion of eels and ropefish differs kinematically from swimming in that longitudinal points of the bodies tend to ‘path-follow’, where any given point of the body is followed by the next posterior point. In the case of eels, axial muscles shift the timing of muscle activation relative to the strain cycle, with fibers activating much later in the cycle on land than when in water. All of these fish demonstrated marked increases in trunk movements when viscosity increased [15] or with terrestriality [6]–[8]. An important distinction from our current findings is that all fish maintained traveling waves of bending and motor activity across environmental transitions [6]–[8], [15].

In aqueous environments, the axial muscle activity of lungfish is propagated posteriorly [15], whereas on the mud surface the activation of muscles in the trunk region conformed more closely to a standing wave. To our knowledge, the only other behaviors in fish and basal tetrapods where ipsilateral blocks of axial muscles are activated simultaneously are during **C**-start escape responses, which are extremely rapid events [22], and the trotting of limbed salamanders [3], [4], [23]. Collectively, previous work has found that the patterns of axial bending during the undulatory locomotion of vertebrates are a complicated result of how the muscles are activated, the mechanical stiffness of the axial structures, and how the body interacts with the mechanical properties of the environment [26], [27]. For example, even when both muscle activity and lateral bending are propagated, a general finding among phylogenetically diverse vertebrates is that these speeds of propagation often differ such that the timing of muscle activity relative to bending often changes along the length of swimming vertebrates (Fig. 4A,B; [4], [10], [11], [15], [23], [28], [29]). Furthermore, the relationship between muscle activity and bending can differ within a species with changes in swimming speed [30] and environment resistance (Fig. 4A vs. B; [7], [10]).

In addition to the considerable variation that can occur with propagated muscle activity and bending, the patterns of bending that occur for standing waves of muscle activity can also be affected by the manner in which the body interacts with the environment. For example, during the initial stage of extremely rapid (<50 ms) escape behaviors fish simultaneously activate all the myomeres on one side of the body, but in some species a lateral wave of bending is propagated posteriorly during this period [31]. Given the many precedents for variable timing between muscle activity and bending, it seems likely that interactions with the surface (such as slipping) could explain why the axial muscle activity of the lungfish on the mud surface conformed to a standing wave pattern but lateral bending did not.

### Trunk-powered vs. tail-powered locomotion

A striking feature of lungfish locomotion that differs between swimming and crawling is the use of the trunk versus tail to power locomotion. With increased viscosity for lungfish [15] or increased swimming speed for eels [32], progressively more muscle segments are recruited anteriorly. Swimming lungfish have higher amplitudes of muscle activity and lateral bending in the tail compared to the trunk region [15], whereas terrestrial locomotion of the lungfish involved higher amplitudes of both muscle activity and lateral bending in the trunk compared to the tail. There is no obvious or discrete alteration in function between trunk and tail during swimming; recruitment of the trunk muscles is not at the exclusion of activity in the caudal region, and the propagation of muscle activity lacks any conspicuous discontinuities as it travels from the trunk to tail regions (Fig. 4B). However, when lungfish move on land muscle activity in the trunk usually occurs without concomitant caudal muscle activity. In fact, the tail often appeared to bend passively rather than as a result of coordinated, detectable muscle activity. Lungfish are elongate, but the caudal region is laterally flattened, with a width less than a third of the diameter of the trunk [15]. Although lungfish may be able to propel themselves by tail movements while suspended in media, the thin, floppy tail is minimally useful for generating propulsive force on land. Furthermore, the standing wave of activity differs from any motor pattern ever observed in the tail during undulatory swimming. An amphibious fish from the Actinopterygii lineage, the walking catfish (*Clarias* spp.), also employs full body bends during terrestrial locomotion; unlike the lungfish, however, this ray-finned fish uses the tail and pectoral appendages for propulsion [24], [25]. Thus, though EMG data are not currently available for this taxon, the distinct lack of tail muscle activity found in the lungfish is not likely to be shared with this organism. Salamanders stepping on land (Fig. 4D; [21]) can have a standing wave in the trunk and a *traveling* wave in the tail, so another intriguing possibility is that the trunk and tail were able to serve as distinct ‘locomotor modules’ [33] in the ancestors of tetrapods.

### Axial function in the evolution of tetrapods

The shift from water to land was a pivotal step in vertebrate evolution, ultimately resulting in the diversity of terrestrial vertebrates present today. Studies of the origins of terrestrial tetrapod locomotion have focused almost entirely on the functional morphology of the limbs of fossil tetrapods and modern analogues [35], [36]. By contrast, the role of axial muscles in early tetrapod locomotion has largely been ignored [but see 37,38] despite the clear importance of axial structures in extant tetrapod locomotion [2], [4], [39]. A reasonable hypothesis is that early tetrapodomorph terrestrial locomotion was powered by axial muscles acting in concert with appendicular structures [16], [38]. Although the movement of modern ray-finned fish like walking catfish are analogous in some respects [24], [25], the axial component of the terrestrial locomotion of both lungfish and salamanders differs in that movement is produced by trunk rather than tail muscles. This fact combined with the evidence that extant lungfish also move their appendicular structures in a coordinated, gait-like pattern [17] is compelling considering that the ancestral state of lungfish appendages and transitional tetrapods was a fleshier, truly ‘lobed’ fin [40], and thus capable of much more weight support. A major evolutionary transition between fishes and tetrapods seems to have been a shift from pectoral appendage dominance to pelvic appendage dominance [34], which renders teleost fish like the walking catfish poor representative models for early tetrapod locomotion. Although the African lungfish are also poor models for the behavior of *limbs* in early tetrapod locomotion, the similarities to amphibian axial function are intriguing.

## Supporting Information

Video S1 Standard speed (30 fps) video of a lungfish moving across a wet clay surface.
(AVI)
